# Prevalence and Regional Differences in Migrated Hips in Danish Children with Cerebral Palsy from 2008 to 2021—A Comparison of Ambulant vs. Non-Ambulant Children

**DOI:** 10.3390/children11080964

**Published:** 2024-08-10

**Authors:** Muhammed Bakhtiyar, Afrim Iljazi, Michael Mørk Petersen, Anders Odgaard, Christian Wong

**Affiliations:** Department of Orthopedic Surgery, Rigshospitalet, 2100 Copenhagen, Denmark

**Keywords:** cerebral palsy, hip displacement, hip dislocation, hip surveillance, GMFCS

## Abstract

Purpose: This study aims to assess the incidence of hip displacement and dislocation (denominated as hip migration) among ambulant and non-ambulant Danish children with cerebral palsy (CP) by estimating their cumulative incidence of migrated hips. A secondary objective is to compare the prevalence across different Danish regions. Methods: Data were obtained from the Danish Cerebral Palsy Follow-Up Program (CPOP) from the years 2008 to 2021. This population-based cohort study included 1388 children with CP (58% male; 42% female) as subjects; aged 0–15 years; with an average age of 5.4 years at their last follow-up. The children were categorized according to their Gross Motor Function Classification System (GMFCS) level into ambulators (GMFCS I–III) and non-ambulators (GMFCS IV–V). The Kaplan–Meier estimator was employed to calculate the cumulative incidence of migrated hips from birth until the date of their last radiographic follow-up. Differences between ambulatory and non-ambulatory children and regional differences were assessed with the Log-rank test. Results: Median radiological follow-up for ambulators was 51 months and 94 months for non-ambulators. The cumulative incidence of hip dislocation was 0.3% (95% CI: 0–0.8%) and 22.0% (95% CI: 9.2–34.8%) for ambulators and non-ambulators, respectively (*p* < 0.0001), whereas the incidence of hip displacement was 21.1% (95% CI: 16.3–25.9%) and 76.7% (95% CI: 68.6–84.7%) for ambulators and non-ambulators, respectively (*p* < 0.0001). There were no significant regional differences in the incidence of hip dislocation among ambulators, but there were significant differences for non-ambulators. Moreover, significant regional differences were detected in hip displacement for both ambulators and non-ambulators. Conclusions: The prevalence of hip migration in Danish children with CP is significantly higher among non-ambulators, who are at an increased risk of hip migration compared to their ambulant counterparts. However, the low frequency of radiographic follow-up for ambulators might cause the incidence of hip migration to be underestimated. This study highlights the necessity of continued targeted surveillance and interventions in Danish non-ambulators.

## 1. Introduction

Cerebral palsy (CP) is one of the most prevalent neurodevelopmental disorders in children, impacting approximately 2 to 3 out of every 1000 births globally [1,2]. CP is commonly characterized by symptoms of muscular dysactivity such as, spasticity, causing motor deficits that can subsequently lead to hip displacement and dislocation (denominated hip migration), which are especially prevalent in the more severely affected children [1,3]. Hip migration may lead to pain, impaired sitting ability, and decreased quality of life when ultimately leading to a complete dislocation of the femoral head out of the acetabulum [4]. Historically, hip migration in CP has been associated with increased muscular overactivity and/or contracture in the hip adductors and flexors, leading to changes in hip joint forces, causing hip migration [5,6,7], but hip migration also occurs in hypotonic CP, indicating other factors such as delayed walking subsequently leading to insufficient skeletal development may play a role [8,9]. It has been shown that ambulatory ability is the main determinant of the progression rate of hip migration, and ambulatory status seems to be more important than adductor spasticity [10,11]. 

Hip surveillance (HS) programs in CP systematically monitor the hip status focusing on, e.g., the longitudinal development of the migration percentage (MP). This enables the early detection of hip migration through longitudinal regular clinical and radiological assessment linked to the risk assessment of the Gross Motor Function Classification System (GMFCS) and chronological age, which subsequently enables timely intervention to avoid or minimize the progression of issues such as migrating hips [12]. Inspired by the Swedish follow-up program for children with CP, the ‘Cerebral Palsy Follow-Up Program’ (CPOP) clinical quality database was commenced in Denmark in 2008 and overseen by the Danish Clinical Quality Program–National Clinical Registries (RKKP), which is responsible for the surveillance of Danish clinical quality registries and quality control [13]. In this database, children diagnosed with CP or presenting equivalent symptoms prior to diagnosis are monitored and evaluated at regular intervals until the age of 15. In addition to the Health Surveillance (HS) program of CPOP, an exhaustive clinical evaluation is conducted. This evaluation includes clinical examinations of gross and fine motor functions, manual ability, muscle tone, passive range of motion, and the use of orthotics and assistive devices, as part of a multidisciplinary effort including specialized physiotherapists, orthopedic surgeons, occupational therapists, and pediatric neurologists. The patient’s own physiotherapist evaluates the ambulatory status, lower extremity range of motion and spasticity, i.e., the hip joint, and specifically the adductor muscles. They assess lower extremity spasticity using the Modified Ashworth Scale (MAS) during the child’s rehabilitation and are responsible for entering their assessments in CPOP. The radiographic assessments of the children’s hips and spine are the responsibility of orthopedic surgeons, who are also responsible for the data entry in CPOP, including MP and acetubular index (AI) The pediatric neurologists evaluate the diagnosis and CP subtype, while the occupational therapist evaluates the child’s hand fine motor skills, joint mobility, and occupational therapy interventions. All of the above-mentioned medical professionals conform to the same national guidelines conducting their examinations within CPOP, and regional CPOP coordinators evaluate the data inputs as quality control. This approach systematically assesses Danish children with CP, potentially enhancing the quality of healthcare [13].

A recent study evaluated the hip status of children using Danish CPOP data with a focus on therapist-led interventions [14]. Another previous study evaluated the overall differences in describing regional differences in the management of CP in school-aged children in Denmark [15]. However, hip migration in Danish children with CP in the context of their ambulation status and potential regional variations as a surrogate parameter for intervention efficacy based on different regional approaches has not been evaluated. Our study aims to examine hip migration between ambulatory and non-ambulatory individuals with the hypothesis that non-ambulatory children experience greater hip migration than ambulatory children. This study is the first in Denmark to assess regional differences in hip migration among children with CP and one of the few to investigate hip migration based on a two-sided ambulatory status [10].

In this exploratory study, we utilized CPOP to assess the cumulative incidence of hip migration stratified by ambulatory status in Danish children with CP. Moreover, we examined the regional variation in hip migration status in Denmark. 

## 2. Materials and Methods

### 2.1. Ethics, Registration, and Data Sharing Plan

The study (H-22046940) was assessed by the Regional Committee on Health Research Ethics, which determined that notification was not necessary when following the Danish Act on Research Studies §2. The study was approved by the Data Protection Agency of the Capital Region of Copenhagen under reference number 2008-41-2240 as stipulated by Danish law J.nr. 2008-41-2240, and we adhered to the relevant guidelines and were approved by the local review board (Privacy).

### 2.2. Study Design and Study Population

**Figure 1:**
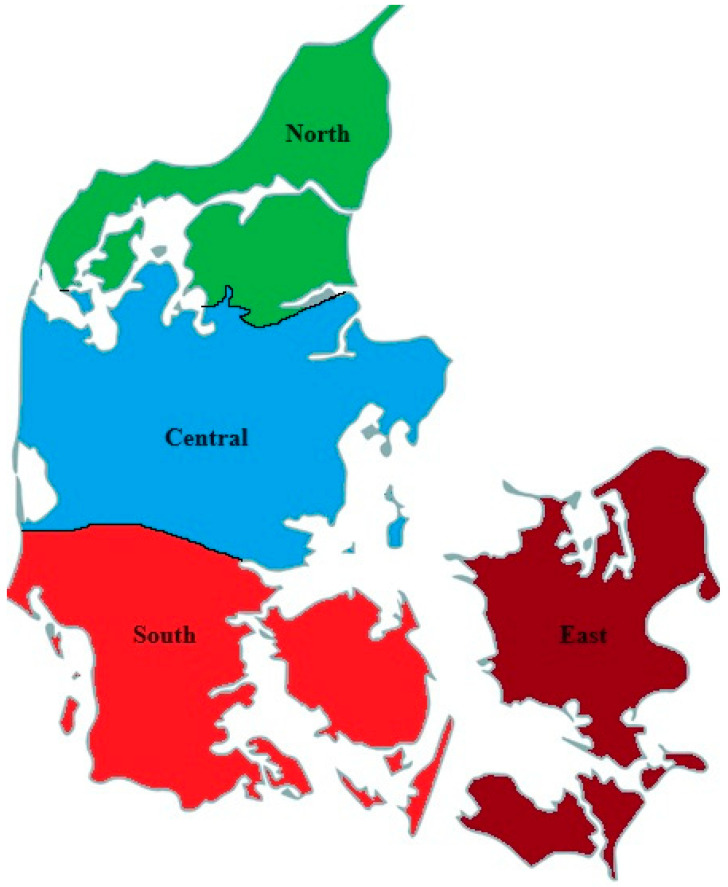
We evaluated the subjects according to regions and divided Denmark into North (consisting of the Northern regions, Central (consisting of the Central region), South (consisting of the Southern region), and East (consisting of the region of Zealand and the Capital region).

The current study is a longitudinal population-based cohort study based on data from the CPOP from database inception in 2008 until 31 December 2021. Healthcare in Denmark operates under a single-payer system. The country is divided into five administrative regions—Capital, Zealand, North, Central, and Southern. Children in the Capital region and the region of Zealand are treated by a unified tertiary healthcare team, which is why we have categorized these children into a single group denominated “East” (Figure 1). The GMFCS score determines radiographic surveillance level and frequency of radiological examination of the hip in CPOP. Children in GMFCS I undergo an X-ray when they reach the age of two or upon enrollment. Children with GMFCS II undergo X-rays between the ages of 2 and 6, or at inclusion and the age of 6. All children in GMFCS III–V have X-rays upon inclusion in the CPOP registry and annually thereafter until the age of seven. All children with hip displacement with MP > 33% are evaluated individually by their treating physician, with additional X-rays upon request and at the discretion of the treating physician. Finally, the treating physician of the children, regardless of their GMFCS level, has the authority to order further X-rays outside of the regular follow-up routine [16].

The study population for this study consisted of Danish children with CP or equivalent symptoms, who were followed up in CPOP and had available data for radiologic examinations of the hip.

### 2.3. Outcomes

The current study is exploratory and describes the following outcomes: age at first entry, age at last follow-up, GMFCS score at first entry, the incidence of hip displacement, defined as the first occurrence of MP ≥ 33%, and dislocation, defined as the first occurrence of MP = 100% [17]. Considering that most children had undergone multiple X-rays, we included all available X-rays for each child in our analysis. We conducted two separate analyses, one for hip displacement and another for hip dislocation. Displacement analysis was performed by evaluating the MP for every consecutive X-ray of each child. A child was identified with hip displacement if their MP ≥ 33% on at least one subsequent X-ray. The time to displacement was defined as the duration from birth to the initial instance of a hip displacement event. In order to avoid duplicating and include the same child numerous times, we only considered the time until the initial occurrence, as this would result in exaggerated results. Children identified with no displacement did not have X-rays with MP ≥ 33%. Subsequently an additional analysis was carried out to evaluate hip dislocation with MP = 100%. The X-rays of each child were then examined. A child was identified with dislocation if their MP = 100% on at least one subsequent X-ray and the time to dislocation was defined as the time until the first occurrence. Children without dislocation were identified as having no reports MP = 100% in any of the available X-rays. The children were followed from the time of birth and until the last radiographic assessment registered in the follow-up program.

### 2.4. Data Analysis

Children were categorized as ambulators (GMFCS I–III) and non-ambulators (GMFCS IV–V) solely based on their GMFCS level [5]. Continuous variables are presented as either means and standard deviations (SD) or median and range. Categorical variables are presented as totals and percentages. The incidence of hip displacement and dislocation was calculated using the Kaplan–Meier estimator, with the data presented as the probability of experiencing an event accompanied by the 95% confidence interval (CI). Differences between groups were assessed using the Log-rank test, with *p* values ≤ 0.05 considered significant. Upon encountering a significant *p* value, we proceeded with pairwise comparisons between each group. We adjusted for multiple testing in the pairwise comparisons using the Bonferroni method. All data analyses were performed using R version 4.3.2 (Posit) [18].

## 3. Results

**Figure 2:**
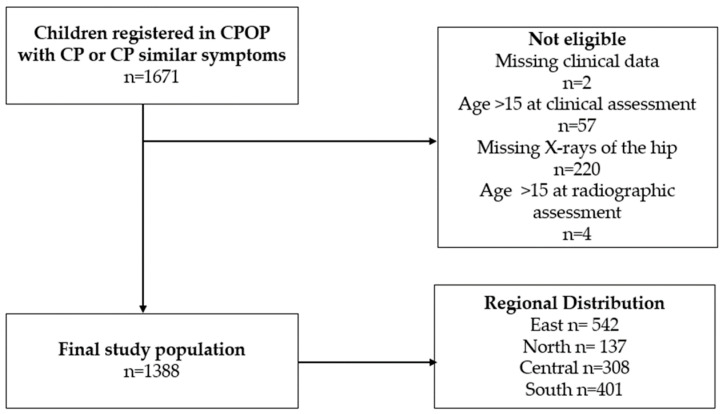
Flowchart illustrating the inclusion process. We screened 1671 children from the Danish ‘Cerebral Parese Opfølgning Program’ (CPOP *). Two hundred and eighty-three subjects were excluded due to the age criteria or missing data. We included 1388 subjects in the study.

We identified 1388 children aged 0–15 years with CP or equivalent symptoms prior to diagnosis, accompanied by radiological data (Figure 2). The study population consisted of 808 boys (58%) and 580 girls (42%), with a mean age of 3.5 years (SD: 2.1) at their initial examination (Table 1). The distribution of GMFCS levels was as follows: 673 children (49%) were classified as GMFCS level I, 255 (18%) as level II, 112 (8%) as level III, 137 (10%) as level IV, and 211 (15%) as level V. Accordingly, 1040 (75%) children were categorized as ambulators, and 348 (25%) as non-ambulators (Table 1). Ambulators underwent a median of 1 X-ray at a median age of 4 years and 4 months (follow-up 51 months), with the number of X-rays ranging from 1 to 13 performed between 9 and 179 months after birth (0.75–15 years) as shown in Table 1. Non-ambulators had a median of 5 X-rays conducted over a mean follow-up of 7 years and 10 months from birth (94 months), with a range of 1 to 21 X-rays performed between 9 and 179 months (0.75–15 years). The mean MP for all children was 19.8% (SD: 18.0), with ambulators at 14.6% (SD: 12.9) and non-ambulators at 35.3% (SD: 21.7). The average AI was 20.3° (SD: 7.3), with ambulators at 19.3° (SD: 5.7) and non-ambulators at 23.5° (SD: 10.2). The number of hip displacement events changed depending on GMFCS level: 25 at level I, 32 at level II, 46 at level III, 68 at level IV, and 134 at level V.

### 3.1. Hip Migration Rates for Ambulators vs. Non-Ambulators

Among ambulating children, 21.1% (CI: 16.3–25.9%) had radiological assessments indicating hip displacement, compared to 76.7% (CI: 68.6–84.7%) among non-ambulators (*p* value < 0.0001), as shown in Table 2. Hip dislocation was observed in 0.3% (CI: 0–0.8%) of ambulating children and 22.0% (CI: 9.2–34.8%) of non-ambulating children (*p* value < 0.0001).

### 3.2. Regional Variation in Hip Migration Status

The incidence of hip displacement among ambulators was 18.1% (CI: 11.4–24.7%) in the East, 25.3% (CI: 17.6–33%) in the South, 26.6% (CI: 9.9–43.3%) in the Central region, and 14.1% (CI: 1.4–26.8%) in the North, with significant regional variation (*p* value < 0.01) attributed to a higher incidence in the South compared to the East (Table 3).

For non-ambulators, the incidence of hip displacement was 71.6% (CI: 59.3–84.0%) in the East, 92.1% (CI: 79.1–100%) in the South, 70.0% (CI: 58.6–81.4%) in the Central region, and 58.1% (CI: 39.2–76.1%) in the North, showing statistically significant variation (*p* value = 0.01) again due to a higher incidence in the South compared to the East (Table 4). Regarding hip dislocation, a single incident was noted among ambulating children in the South, corresponding to a regional incidence of 0.9% (CI: 0–2.6%) (Table 3). Among non-ambulating children, dislocation rates were 15.4% (CI: 3.3–27.5%) in the East, 24.4% (CI: 4.6–44.1%) in the South, 27.0% (CI:11.2–46.8%) in the Central region, and 0% in the North (*p* = 0.01), with the regional disparity explained by a difference between the Central region and the North (*p* = 0.036) (Table 4).

## 4. Discussion

In this study, we have performed a longitudinal investigation of hip migration in Danish children with CP from data in CPOP. Notably, our findings reveal a pronounced disparity in the incidence of migrated hips between ambulatory and non-ambulatory children, underscoring the significant role of ambulation (as indicated by the GMFCS level) for the prognosis of hip migration in children with CP [1]. This dichotomy aligns with the existing literature, which consistently shows a higher risk of hip migration in children with higher GMFCS levels, emphasizing the importance of targeted therapist- and orthopedic-led interventions for non-ambulators [3,4]. Terjesen (2006) demonstrated the role of ambulation in a smaller (*n* = 76) population, referred to an orthopedic department, and thus a selected population, and we can confirm his finding in our larger and ‘unselected’ population [10].

We found regional significant differences in hip displacement and dislocation for non-ambulators in one Danish region. We speculate if this is caused by a difference in therapist-led or orthopedic interventions. Krarup et al. (2023) found that half of the Danish children with hip displacement received intensive therapist-led interventions with standing aids 5–6 days per week and joint range of motion, postural stability, and muscle strength sessions in general. However, they did not explore regional variations in these interventions [14]. Previous studies examining botulinum toxins, physiotherapy, and hip abduction braces indicated no effect on hip status in a prospective randomized design [19], but Poutney et al. in 2001 [20] found that therapist-led interventions utilizing multiple postural support systems were found to significantly reduce the incidence of hip subluxation (MP > 33%) in children with cerebral palsy compared to those who did not use these systems when examined in a retrospective design, as well as in 2009 in a prospective study compared to a control group [21]. They investigated therapist-led therapies in children under 18 months with cerebral palsy (CP) and the use of postural support equipment. The study indicated that there were significant changes in the rate of hip subluxation. The findings of both studies indicate for specific therapist-led interventions as supported standing can impact chances of hip migration when applied in infancy.

Rackauskaite et al. (2015) found regional differences in certain therapist-led interventions, which might account for these regional differences in hip displacement status. There are as yet no data on regional differences in orthopedic hip-related interventions for children with CP, but Rackauskaite et al. (2015) found a higher rate of orthopedic surgery in GMFCS V in the Capital region in general, which might also account for some of these differences in hip displacement and dislocation [15]. However, we also speculate if this might be influenced by ‘alternative therapies’ [22], where some of these do not adhere to surgical interventions, leading to the parental refusal of offered orthopedic interventions, and some of these alternative therapy centers are situated geographically in the region with a significantly higher hip migration [23]. However, these unexplained regional differences in hip migration underscore the necessity to investigate the demography of regional differences in orthopedic-led hip-related interventions. If the prevention of hip migration is indeed beneficial, this would highlight the benefits of a uniform healthcare strategy similar to countries with established CP monitoring programs [12,24].

### Study Limitations

A limitation in this longitudinal study is the potential occurrence of “silent hips” where hip migration occurs in children with CP, GMFCS I–II without early clinical symptoms, and GMFCS III–V after the age of 7 with late hip migration [25,26]. This condition poses a challenge to early detection, as it may not be identified until late dislocation, which can only be resolved by salvage procedures. Clinical symptoms and scheduled radiographic follow-ups may not promptly identify these silent progressions [24]. However, ambulators have a significantly lower cumulative incidence of hip dislocation than their non-ambulant counterparts [1]. In this study, we might underestimate the incidence of hip dislocation in ambulators, given that these individuals might not receive as frequent or thorough monitoring for hip dislocation as non-ambulators, thereby potentially missing early signs of “silent” hip dislocations. Furthermore, in our study, there is the potential for selection bias inherent in the study’s design. The study’s population was derived from the Danish CPOP, which involves a longer median radiographic follow-up for non-ambulators (94 months) compared to ambulators (51 months). The difference in monitoring intensity could influence the observed incidence rates of hip dislocation, with a preference for a relatively higher ‘detection rate’ of hip migration in non-ambulators. This discrepancy could lead to a selection bias, where non-ambulators are more likely to be closely monitored and thus have their hip dislocations detected and documented in the study, compared to their ambulant counterparts.

The study’s data were sourced from the national CPOP database, which could have inherent biases. Having numerous examiners with various experience levels could result in ‘measurement bias’ in radiological assessments. Fajac et al. (2004) demonstrated that an experienced rater usually shows a variability of around 8% for radiological MP assessments. Less-experienced observers had median intra-observer differences ranging from 3.2% to 3.6% and inter-observer differences ranging from 3.3% to 5% [27], thus resulting in measurement bias.

In general, our findings on the positive role of ambulation for hip migration in a large Danish cohort as well as the regional variations in hip migration incidence rates might be influenced by the frequency of radiographic monitoring, the patients’ acceptance of interventions, and the availability of therapist- and orthopedic-led interventions. We acknowledge that our study is limited by not including these surgical and non-surgical treatments for migrated hips. When we carefully consider the wider implications of our findings, we find that the high occurrence of displaced and dislocated hips in non-ambulators justifies the necessity for continuous monitoring and underscoring the significance of early and proactive intervention techniques if indeed the prevention of hip migration is beneficial [24]. Our study indicates that the HS of the Danish CPOP can identify non-ambulatory children prone to hip migration and detect significant regional differences. We can confirm that ambulation is an important factor for hip migration. Future studies should address other related factors for hip migration, and as a previous study comprehensively explored the therapist-led interventions [14], they should explore the role of especially orthopedic-led surgical interventions to address the regional differences in hip migration and potentially improve treatment strategies across all regions.

## 5. Conclusions

In Danish children with CP, our study found that the incidence of hip migration is significantly more frequent in non-ambulators, who face a larger risk of hip migration compared to ambulant individuals. These findings are consistent with the existing literature. Our analysis also uncovered significant regional differences in the prevalence of hip migration among Danish children with CP, emphasizing the necessity for additional research on its underlying causes. These changes may affect the overall occurrence rates and provide information for particular interventions. Future research ought to focus on investigating these regional disparities to enhance the management and prevention of hip migration in children with cerebral palsy across different regions.

## Figures and Tables

**Table 1:** Baseline characteristics of the complete cohort.

	Total	Ambulators	Non-Ambulators
Patients	1388	1040 (75%)	348(25%)
GMFCS level		I: 673 (49%) II: 255 (18%) III: 112 (8%)	
			IV: 137 (10%) V: 211 (15%)
Sex			
Male	808 (58%)	617 (59%)	191 (55%)
Female	580 (42%)	423 (41%)	157 (45%)
Age in years			
At entry	3.5 ± 2.1	3.5 ± 2.1	3.3 ± 2.2
At last observation	5.4 ± 3.1	4.8 ± 2.7	7.3 ± 3.2
Radiographic data			
Total number of X-rays	2 (1–21)	1 (1–13)	5 (1–21)
Follow-up in months	58 (9–179)	51 (9–179)	94 (9–179)
MP	19.8 ± 18.0	14.6 ± 12.9	35.3 ± 21.7
AI	20.3 ± 7.3	19.3 ± 5.7	23.5 ± 10.2
Number of hip displacement events		I: 25 II: 32 III: 46	
			IV: 68 V: 134

**Table 2:** Hip displacement and dislocation rates in ambulators vs. non-ambulators.

	Ambulators	Non-Ambulators	*p* Value
Hip displacement	21.1% (16.3–25.9%) *n* = 103	76.7% (68.6–84.7%) *n* = 202	<0.0001
Hip dislocation	0.3% (0–0.8%) *n* = 1	22.0% (9.2–34.8%) *n* = 32	<0.0001

**Table 3:** Hip displacement and dislocation rates distribution along Danish regions in ambulators.

Outcome	East *n* = 431	South *n* = 296	Central *n* = 213	North *n* = 100	*p* Value
Hip displacement	18.1% (11.4–24.7%)	25.3% (17.6–33%)	26.6% (9.9–43.3%)	14.1% (1.4–26.8%)	0.01 ^1^
Hip dislocation	0.0%	0.9% (0.0–2.6%)	0.0%	0.0%	0.5

^1^ Pairwise Log-rank test adjusted for multiple testing showed a difference between South and East (*p* = 0.03) but not between any of the other regions.

**Table 4:** Hip displacement and dislocation rates distribution along Danish regions in non-ambulators.

Outcome	East *n* = 111	South *n* = 105	Central *n* = 95	North *n* = 37	*p* Value
Hip displacement	71.6% (59.3–84.0%)	92.1% (79.1–100%)	70.0% (58.6–81.4%)	58.1% (39.2–76.1%)	0.02 ^1^
Hip dislocation	15.4% (3.3–27.5%)	24.4% (4.6–44.1%)	27.0% (11.2–46.8%)	0.0%	0.01 ^2^

^1^ Pairwise Log-rank test adjusted for multiple testing showed a difference between South and East (*p* = 0.024) but not between any of the other regions. ^2^ Pairwise Log-rank test adjusted for multiple testing showed a difference between Central and North (*p* = 0.036) but not between any of the other regions.

## Data Availability

Restrictions apply to the availability of these raw data. Data were obtained from RKKP at “https://www.rkkp.dk/ (accessed on 1 October 2022)”.
